# Mechanisms driving plant functional trait variation in a tropical forest

**DOI:** 10.1002/ece3.7256

**Published:** 2021-03-13

**Authors:** Florian Hofhansl, Eduardo Chacón‐Madrigal, Åke Brännström, Ulf Dieckmann, Oskar Franklin

**Affiliations:** ^1^ International Institute for Applied Systems Analysis Laxenburg Austria; ^2^ Escuela de Biología Universidad de Costa Rica San José Costa Rica; ^3^ Department of Mathematics and Mathematical Statistics Umeå University Umeå Sweden; ^4^ Department of Evolutionary Studies of Biosystems The Graduate University for Advanced Studies (Sokendai) Hayama Japan

**Keywords:** Biodiversity, climate change, Costa Rica, plant functional traits, tropical forest

## Abstract

Plant functional trait variation in tropical forests results from taxonomic differences in phylogeny and associated genetic differences, as well as, phenotypic plastic responses to the environment. Accounting for the underlying mechanisms driving plant functional trait variation is important for understanding the potential rate of change of ecosystems since trait acclimation via phenotypic plasticity is very fast compared to shifts in community composition and genetic adaptation. We here applied a statistical technique to decompose the relative roles of phenotypic plasticity, genetic adaptation, and phylogenetic constraints. We examined typically obtained plant functional traits, such as wood density, plant height, specific leaf area, leaf area, leaf thickness, leaf dry mass content, leaf nitrogen content, and leaf phosphorus content. We assumed that genetic differences in plant functional traits between species and genotypes increase with environmental heterogeneity and geographic distance, whereas trait variation due to plastic acclimation to the local environment is independent of spatial distance between sampling sites. Results suggest that most of the observed trait variation could not be explained by the measured environmental variables, thus indicating a limited potential to predict individual plant traits from commonly assessed parameters. However, we found a difference in the response of plant functional traits, such that leaf traits varied in response to canopy‐light regime and nutrient availability, whereas wood traits were related to topoedaphic factors and water availability. Our analysis furthermore revealed differences in the functional response of coexisting neotropical tree species, which suggests that endemic species with conservative ecological strategies might be especially prone to competitive exclusion under projected climate change.

## Graphical abstract

We applied a statistical technique to analyze the relative amount of variation within plant functional traits associated with (a) environmental heterogeneity and spatial distance, as well as (b) bioclimatic variables in response to canopy‐light regime and soil nutrient availability.

We found that plant functional reaction norms to bioclimatic variables reflect differences in the eco‐evolutionary trajectory and associated ecological life‐history strategy between congeneric pairs of widespread and coexisting endemic neotropical tree species.

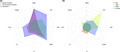

## 1 INTRODUCTION

In general, variation of plant functional characteristics should enhance a plant's ability to cope with shifts in the local environment as species with higher trait variability should exhibit greater trait–environment matching than less variable species (Mitchell et al., 2016). Such trait variation includes plasticity in a species’ characteristics that enhances its ability to quickly respond to environmental changes (Fox et al., 2019), as well as genotypic adaptation (evolution) in response to environmental variation over longer timespans (Murren et al., 2015). Consequently, species with a high degree of trait plasticity have been found much more likely to succeed in a given environment (Hulme, 2008) and, vice versa, species showing low plasticity have been found more vulnerable to changing environmental conditions (Sides et al., 2014). Hence, accounting for the different underlying mechanisms driving trait variation, and in particular to differentiate plasticity from other mechanisms of trait variation, is important for understanding and accurate modeling of vegetation dynamics (Franklin et al., 2020).

The underlying mechanisms driving trait variation in tropical forests are associated with multiple environmental drivers as factors shaping species composition, and thus determining associated plant functional traits, have been reported to shift across latitudinal and altitudinal gradients (Ackerly & Cornwell, 2007). For instance, it has been found that across larger spatial scales abiotic factors, such as temperature and precipitation, are key determinants of ecosystem processes (Cleveland et al., 2011; Taylor et al., 2017). However, at smaller spatial scales other biotic factors, such as competition among coexisting tree species, strongly affect ecosystem structure and functioning via the composition of the local species pool (Fauset et al., 2012; Taylor et al., 2015). Accordingly, it has been shown that competition can have equally strong impacts on trait expression as the dominant abiotic driver (Albert et al., 2010; Le Bagousse Pinguet et al., 2015; Violle et al., 2012), which further highlights that it is crucial to account for different components driving plant functional trait variation (Jung et al., 2010). So far, most studies have been assessing mean‐species’ trait values compiled from published datasets comprising global observations (Freschet et al., 2011; Kraft et al., 2008; Swenson & Enquist, 2007) and thus have been unable to differentiate plant functional responses to multiple and interactive controlling factors (Ackerly & Cornwell, 2007; Sides et al., 2014).

Here, we aimed to differentiate the underlying mechanisms controlling plant functional trait variation in a tropical forest and to quantify respective contributions of multiple and interrelated environmental factors. We compiled a trait dataset from in‐situ measurements of the following plant functional traits: (a) wood density, as an important part of the wood‐economics spectrum (Chave et al., 2009) associated with drought tolerance and shade tolerance; (b) maximum plant height, as a strategy to enhance light exposure and linked to drought vulnerability (Rowland et al., 2015); (c) leaf area, (d) leaf thickness, and (e) specific leaf area, which are associated with light capture; (f) leaf dry mass, (g) leaf nitrogen content, and (h) leaf phosphorus content, which are included in the leaf‐economics spectrum (Wright et al., 2004) and are related to local soil water and nutrient availability.

For each of the plant functional traits investigated in this study, we aimed to differentiate respective components of trait variation, in particular, the amount of phenotypic plasticity versus other components, including genetic adaptation and species turnover between sample sites. Although phenotypic plasticity is influenced by many different factors, here we focus on a particularly relevant aspect with respect to climate change (i.e., trait plasticity driven by environmental variation). We evaluated respective components of trait variation based on the underlying driving factors, that is, local environmental heterogeneity independent of geographic distance among study sites (i.e., the pure environmental factor), spatial distance between sample sites, while accounting for environmental heterogeneity among study sites (i.e., the pure spatial factor), and unknown factors not accounted for in the analysis (i.e., the unexplained variation factor). In addition, we tested the hypothesis that coexisting neotropical tree species differ in the degree of trait plasticity due to differences in the eco‐evolutionary trajectory between range‐restricted and more widespread species.

## 2 MATERIAL AND METHODS

### 2.1 Study region

The study was conducted in tropical lowland forests located between 50 and 450 m a. s. l. in the Área de Conservación Osa (ACOSA) at the Pacific slope of southwestern Costa Rica (08.6°N, 83.2°W). The region was declared a biodiversity hotspot with 700 tree species among 2,369 species of ferns, fern allies, and flowering plants recorded in total (Quesada et al., 1997). The terrain is characterized by parent material originating from the Cretaceous, Tertiary, and Quaternary (i.e., basalt, alluvium and sediment) and is divided into six different landforms (i.e., denudational, volcanic, alluvial, structural, littoral, tectonic) and four soil orders (i.e., Entisols, Inceptisols, Mollisols, and Ultisols (Lobo, 2016)). The dominating, highly weathered, strongly acidic Ultisols on ridges and upper slopes are replaced by younger, moderately weathered Inceptisols in ravines and lower slopes and little developed Mollisols in fluvial deposits (Lobo, 2016). Starting in 1997, daily climatologic data for temperature and precipitation are available from a nearby meteorological weather station located at La Gamba field station: https://www.lagamba.at/en/tropical‐field‐station/scientific‐data‐of‐the‐golfo‐dulce‐region/. Mean annual precipitation for the period 1998–2008 was 5,892 mm, with no month receiving less than 180 mm on average. The rainy season usually lasts from April to December, and the driest months are January to March. Mean annual temperature for the period 1998–2008 was 28.0°C and ranged between 23.7°C and 33.7°C (Weissenhofer et al., 2008).

### 2.2 Environmental variation among sampling sites

In order to account for environmental variation among sampling sites and associated effects on trait variation among congeneric tree species, we measured the slope of the forest stand (using a clinometer) and estimated crown exposure to light using an index from 0 to 5. Moreover, we took geographical coordinates using a GPS device (Garmin 60 CSX, with a mean relative standard error of 6 m). Based on these coordinates, we extracted bioclimatic variables (at a resolution of ~ 1 km^2^) from Worldclim (Hijmans et al., 2005), including annual mean temperature, mean diurnal temperature range, isothermality (ratio of day‐to‐night temperature oscillation to summer‐to‐winter temperature oscillation), annual precipitation, precipitation seasonality, and precipitation during warmest quarter.

### 2.3 Selection of tropical tree species and plant functional traits

A full description of tropical tree species selected for sampling of plant functional traits has been reported in a foregoing study (Chacón‐Madrigal et al., 2018a). Briefly, we selected 34 tree species from 14 genera and grouped them into pairs of congeneric species (Table 1). Each congeneric pair comprised one narrowly endemic species (either restricted to the central and southern Pacific slope of Costa Rica or, in some cases, reaching western Panama or the Caribbean slope in Costa Rica), and one species distributed more widely. From each of the ten selected tree individuals per species (*n* = 335), we collected five fully expanded, mature leaves with no signs of damage and one wood core from each tree. For each tree, we determined wood density, quantified by wood specific gravity (WSG) on a collected wood core, and measured total plant size, that is, tree height (Height). For each leaf of each tree, we analyzed four functional traits: leaf area (LA), leaf thickness (LT), leaf dry‐matter content (LDMC), and specific leaf area (SLA) according to standard protocols (Pérez‐Harguindeguy et al., 2013). On a pooled leaf sample per individual, we further measured leaf nitrogen content (LNC) and leaf phosphorus content (LPC). LNC was measured by dry combustion using an auto analyzer (Rapid Exceed, Elementar, Langenselbold, Germany), and LPC was determined by acid digestion and inductively coupled plasma‐optical emission spectroscopy (ICP‐OES) using a spectrometer Optima 8,300 (Perkin Elmer, Waltham, US) at the laboratory of the Agronomic Research Center (Centro de Investigaciones Agronómicas) of the University of Costa Rica (UCR).

**TABLE 1:** Variation in eight plant functional traits

Family name	Species name	Range class	*n*	WSG ± SE	Height ± SE	SLA ± SE	LA ± SE	LT ± SE	LDMC ± SE	LNC ± SE	LPC ± SE
Annonaceae	*Guatteria amplifolia Triana & Planch*.	widespread	10	0.42 ± 0.01	8.06 ± 0.91	130.97 ± 4.12	294.90 ± 27.10	0.23 ± 0.01	419.01 ± 7.75	1.67 ± 0.03	0.06 ± 0.00
Annonaceae	*Guatteria chiriquiensis R. E. Fr*.	endemic	9	0.40 ± 0.01	11.89 ± 0.93	165.52 ± 13.32	111.03 ± 4.30	0.27 ± 0.01	310.12 ± 14.73	2.32 ± 0.12	0.10 ± 0.01
Annonaceae	*Guatteria pudica N.Zamora & Maas*	endemic	16	0.53 ± 0.01	9.88 ± 1.02	152.77 ± 7.44	96.51 ± 7.88	0.33 ± 0.01	304.82 ± 6.78	1.90 ± 0.04	0.08 ± 0.00
Annonaceae	*Guatteria rostrata Erkens & Maas*	widespread	10	0.41 ± 0.01	11.14 ± 1.54	153.89 ± 3.52	179.31 ± 15.20	0.21 ± 0.01	344.58 ± 7.77	2.07 ± 0.07	0.10 ± 0.01
Annonaceae	*Unonopsis osae Maas & Westra*	endemic	10	0.61 ± 0.01	5.45 ± 0.44	168.09 ± 7.10	102.18 ± 8.29	0.20 ± 0.00	435.68 ± 10.32	1.82 ± 0.05	0.07 ± 0.01
Annonaceae	*Unonopsis theobromifolia N. Zamora & Poveda*	widespread	10	0.51 ± 0.01	11.25 ± 1.46	160.57 ± 5.54	256.30 ± 12.40	0.31 ± 0.01	429.76 ± 6.67	1.95 ± 0.04	0.09 ± 0.01
Araliaceae	*Dendropanax arboreus (L.) Decne. & Planch*.	widespread	10	0.43 ± 0.01	7.89 ± 0.54	145.92 ± 5.18	129.59 ± 10.73	0.29 ± 0.01	284.37 ± 5.53	1.53 ± 0.06	0.07 ± 0.00
Araliaceae	*Dendropanax ravenii M. J. Cannon & Cannon*	endemic	10	0.54 ± 0.01	2.59 ± 0.27	220.19 ± 6.13	77.08 ± 5.59	0.22 ± 0.01	232.35 ± 4.75	1.79 ± 0.05	0.08 ± 0.01
Boraginaceae	*Cordia cymosa (Donn. Sm.) Standl*.	widespread	8	0.26 ± 0.02	11.46 ± 1.16	159.53 ± 13.47	501.48 ± 22.91	0.39 ± 0.02	290.21 ± 10.00	2.05 ± 0.09	0.12 ± 0.01
Boraginaceae	*Cordia liesneri J. S. Mill*.	endemic	9	0.55 ± 0.02	3.96 ± 0.29	137.97 ± 9.65	315.31 ± 32.57	0.31 ± 0.01	349.54 ± 17.16	1.87 ± 0.08	0.06 ± 0.00
Burseraceae	*Protium panamense (Rose) I. M. Johnst*.	widespread	8	0.49 ± 0.03	11.58 ± 1.11	123.17 ± 6.04	1,156.28 ± 156.65	0.23 ± 0.03	418.82 ± 6.93	1.69 ± 0.09	0.11 ± 0.01
Burseraceae	*Protium pecuniosum D. C. Daly*	endemic	10	0.53 ± 0.02	14.04 ± 1.38	125.52 ± 5.11	1645.36 ± 101.30	0.19 ± 0.00	433.66 ± 10.25	1.85 ± 0.08	0.10 ± 0.01
Clusiaceae	*Chrysochlamys glauca (Oerst. ex Planch. & Triana) Hemsl*.	widespread	10	0.57 ± 0.02	5.73 ± 0.75	228.97 ± 9.19	53.84 ± 4.38	0.25 ± 0.01	195.21 ± 5.06	2.09 ± 0.16	0.10 ± 0.01
Clusiaceae	*Chrysochlamys skutchii Hammel*	endemic	9	0.63 ± 0.02	7.10 ± 0.47	102.18 ± 8.58	226.22 ± 35.19	0.37 ± 0.02	286.44 ± 8.58	1.34 ± 0.13	0.06 ± 0.01
Clusiaceae	*Garcinia aguilari Hammel*	endemic	10	0.79 ± 0.01	9.79 ± 1.35	79.69 ± 3.63	495.55 ± 69.72	0.38 ± 0.01	428.95 ± 7.37	1.24 ± 0.04	0.07 ± 0.01
Clusiaceae	*Garcinia magnifolia (Pittier) Hammel*	widespread	10	0.75 ± 0.01	16.99 ± 1.95	66.41 ± 3.87	444.92 ± 26.95	0.62 ± 0.02	369.33 ± 11.72	1.37 ± 0.03	0.06 ± 0.00
Euphorbiaceae	*Sapium allenii Huft*	endemic	11	0.36 ± 0.02	11.88 ± 1.56	185.81 ± 22.24	190.86 ± 28.12	0.26 ± 0.02	233.38 ± 21.63	2.42 ± 0.18	0.23 ± 0.03
Euphorbiaceae	*Sapium glandulosum (L.) Morong*	widespread	10	0.37 ± 0.01	8.90 ± 0.78	156.57 ± 15.28	84.79 ± 5.16	0.26 ± 0.01	288.29 ± 11.98	2.12 ± 0.07	0.19 ± 0.03
Fabaceae	*Inga skutchii Standl*.	endemic	10	0.68 ± 0.02	4.97 ± 0.69	236.33 ± 12.63	138.55 ± 11.30	0.16 ± 0.01	387.73 ± 11.22	3.07 ± 0.08	0.11 ± 0.01
Fabaceae	*Inga spectabilis (Vahl) Willd*	widespread	9	0.52 ± 0.02	6.78 ± 0.32	99.51 ± 4.73	581.74 ± 47.89	0.35 ± 0.02	388.09 ± 11.17	2.80 ± 0.15	0.12 ± 0.01
Lauraceae	*Ocotea mollifolia Mez & Pittier*	widespread	10	0.42 ± 0.02	7.94 ± 0.48	147.55 ± 10.56	269.38 ± 21.48	0.32 ± 0.01	343.69 ± 10.10	1.94 ± 0.07	0.07 ± 0.00
Lauraceae	*Ocotea rivularis Standl. & L. O. Williams*	endemic	9	0.37 ± 0.01	9.47 ± 0.75	108.44 ± 4.43	476.92 ± 49.80	0.32 ± 0.01	320.47 ± 8.82	1.96 ± 0.12	0.10 ± 0.01
Melastomataceae	*Miconia dissitinervia Kriebel, Almeda & A. Estrada*	endemic	11	0.61 ± 0.01	4.77 ± 0.28	165.19 ± 4.97	199.10 ± 19.41	0.39 ± 0.01	340.76 ± 5.72	1.60 ± 0.05	0.07 ± 0.01
Melastomataceae	*Miconia donaeana Naudin*	widespread	10	0.55 ± 0.01	7.69 ± 0.80	200.58 ± 9.16	174.91 ± 14.65	0.29 ± 0.01	336.58 ± 7.56	1.92 ± 0.05	0.07 ± 0.00
Melastomataceae	*Miconia osaensis Aguilar, Kriebel & Almeda*	endemic	10	0.57 ± 0.01	10.95 ± 1.30	85.94 ± 2.55	155.83 ± 22.43	0.39 ± 0.01	403.81 ± 9.47	1.46 ± 0.05	0.06 ± 0.00
Melastomataceae	*Miconia trinervia (Sw.) D. Don ex Loudon*	widespread	10	0.51 ± 0.01	7.06 ± 0.57	131.34 ± 7.64	246.38 ± 18.34	0.22 ± 0.01	288.52 ± 11.33	1.97 ± 0.06	0.08 ± 0.01
Primulaceae	*Ardisia compressa Kunth*	widespread	9	0.58 ± 0.02	11.60 ± 2.07	167.98 ± 7.41	53.17 ± 3.66	0.25 ± 0.01	249.92 ± 9.35	1.87 ± 0.06	0.10 ± 0.01
Primulaceae	*Ardisia dunlapiana P. H. Allen*	endemic	10	0.84 ± 0.01	8.54 ± 1.01	118.64 ± 3.56	38.04 ± 2.78	0.30 ± 0.01	306.92 ± 9.85	1.17 ± 0.02	0.05 ± 0.00
Rubiaceae	*Faramea occidentalis (L.) A. Rich*.	widespread	11	0.63 ± 0.01	6.59 ± 0.75	160.95 ± 3.98	49.63 ± 3.77	0.29 ± 0.01	405.56 ± 3.45	1.43 ± 0.06	0.06 ± 0.00
Rubiaceae	*Faramea permagnifolia Dwyer ex C. M. Taylor*	endemic	12	0.62 ± 0.02	3.42 ± 0.19	110.39 ± 5.22	364.84 ± 21.26	0.37 ± 0.01	301.95 ± 8.76	1.23 ± 0.06	0.05 ± 0.00
Sapotaceae	*Pouteria lecythidicarpa P. E. Sa ´nchez & Poveda*	endemic	10	0.85 ± 0.01	7.49 ± 0.56	74.05 ± 4.14	1,084.26 ± 163.68	0.25 ± 0.01	413.20 ± 12.58	1.61 ± 0.10	0.08 ± 0.01
Sapotaceae	*Pouteria subrotata Cronquist*	widespread	8	0.77 ± 0.02	14.99 ± 1.67	125.95 ± 5.25	208.22 ± 11.45	0.18 ± 0.00	429.58 ± 14.88	2.29 ± 0.13	0.10 ± 0.01
Sapotaceae	*Pouteria torta (Mart.) Radlk*.	widespread	10	0.86 ± 0.02	12.45 ± 2.16	122.88 ± 6.61	252.83 ± 38.69	0.23 ± 0.02	465.45 ± 8.43	1.73 ± 0.07	0.06 ± 0.01
Sapotaceae	*Pouteria triplarifolia C. K. Allen ex T. D. Pennington*	endemic	6	0.73 ± 0.02	8.50 ± 1.69	119.68 ± 2.10	291.31 ± 31.92	0.21 ± 0.00	471.84 ± 7.36	1.38 ± 0.09	0.07 ± 0.00

Note

Among 34 tree species sampled in tropical lowland forests located in southwestern Costa Rica. Values represent the mean ± the standard error of sampled tree individuals with the actual number of samples indicated in the column titled *n*. Species are classified as being either widespread or endemic

Abbreviations: Height, plant height; LA, leaf area; LMDC, leaf dry‐matter content; LNC, leaf nitrogen content; LPC, leaf phosphorous content; LT, leaf thickness; SLA, specific leaf area; WSG, wood specific gravity, i.e., wood density.

### 2.4 Theory and assumptions

While functional trait variation and phenotypic plasticity are governed by complex interactions among genetic and environmental factors, here we address solely the component of trait plasticity driven by environmental variation. Our approach does not separate plasticity from ontogenetic effects or possible micro‐scale adaptation (Brousseau et al., 2015; Richardson et al., 2014), as this was not feasible based on the available dataset. Here, we focus on trait variation among sampled tree individuals, while accounting for species and intra‐specific genetic differences, both of which are influenced by the environment but will additionally be affected by other factors, such as spatial distance between individuals. We here applied a statistical technique to separate environment‐driven plasticity from other sources of trait variation (i.e., spatial distance effects) based on the observed variation of plant functional traits sampled from tree individuals occuring at different locations in the study region. We tried to avoid ontogenetic effects on trait variation by selecting only mature individuals (classified as such based on their diameter at breast height) and accounted for species phylogeny and differences in range size among coexisting widespread and congeneric endemic tree species by analyzing species mean values.

### 2.5 Statistical analysis

Statistical analyses were performed using the R statistical software environment and respective packages “cati,” “ecodist,” “fmsb,” “lme4,” “vegan” (R Core Team, 2018).

We performed a principal component analysis (PCA) relating the investigated eight plant functional traits to in‐situ observed environmental variables (slope of the forest stands and estimated crown exposure to light). In addition, for unmeasured climatic variables we extracted Worldclim bioclimatic variables (i.e., annual mean temperature, mean diurnal temperature range, isothermality (ratio of day‐to‐night temperature oscillation to summer‐to‐winter oscillation), annual precipitation, precipitation seasonality, and precipitation of warmest quarter). We then combined these environmental variables after normalization by means of z‐scores (first ordination axis explaining 86% of the variation) to characterize the mesoclimatic environment of the sampled plant functional traits and plotted respective factor loadings for mean annual temperature and relative humidity (“Climate”), soil clay, sand and silt content (“Soil”), topography (“Slope”), and canopy‐light index (“Light”).

We used linear mixed effects models to test for significant factors driving plant functional trait variation, while accounting for random effects due differences in sites, plot location, species composition, and random factors: [lme(factor ~ 1, random=~1|Locality/Plot/Species/UID)]. To furthermore account for spatial autocorrelation between sample sites and taxonomic constraints among species, we applied multiple regression on distance matrices (MRM), which has been used to disentangle the influence of space and environmental factors in ecological data (Lichstein, 2006) and to relate phylogenetic or functional beta diversity to spatial and environmental distance (Swenson, 2014). In this study, we used MRM to relate a response distance matrix (∂_Y_) with respective distance matrices accounting for environmental, spatial, and interactive effects. To this end, we calculated correlation coefficients between distance matrices of plant functional traits (∂_T_), environmental factors (∂_E_), and geographic distance (∂_S_), and partitioned the total observed variation into components of pure environment (E), pure spatial distance (S), and spatial distance–environment interaction (SxE), respectively. This approach allowed to quantify the relative contribution of factors driving plant functional trait variation due to (a) the correlation between trait distance matrix and environmental distance matrix (while accounting for spatial autocorrelation), (b) the correlation between trait distance matrix and spatial distance matrix (while accounting for environmental heterogeneity), and (c) the correlation between the geographic distance matrix and environmental distance matrix).

We used variance partitioning to quantify respective amounts of variation for each of the plant functional traits, and environmental controlling factors, applied one‐sided Wilcoxon signed‐rank test to assess differences in trait medians between the congeneric pairs of endemic and widespread tropical tree species, and tested for phylogenetic constraints on trait variance for each of the eight plant functional traits, that is, wood specific gravity, i.e., wood density (WSG), plant height (Height), specific leaf area (SLA), leaf area (LA), leaf thickness (LT), leaf dry‐matter content (LMDC), leaf nitrogen content (LNC), leaf phosphorous content (LPC), by constructing a taxonomic dendrogram for the 34 tropical tree species investigated in this study.

## 3 RESULTS

### 3.1 Drivers of plant functional trait variation in tropical forests

**FIGURE 1:**
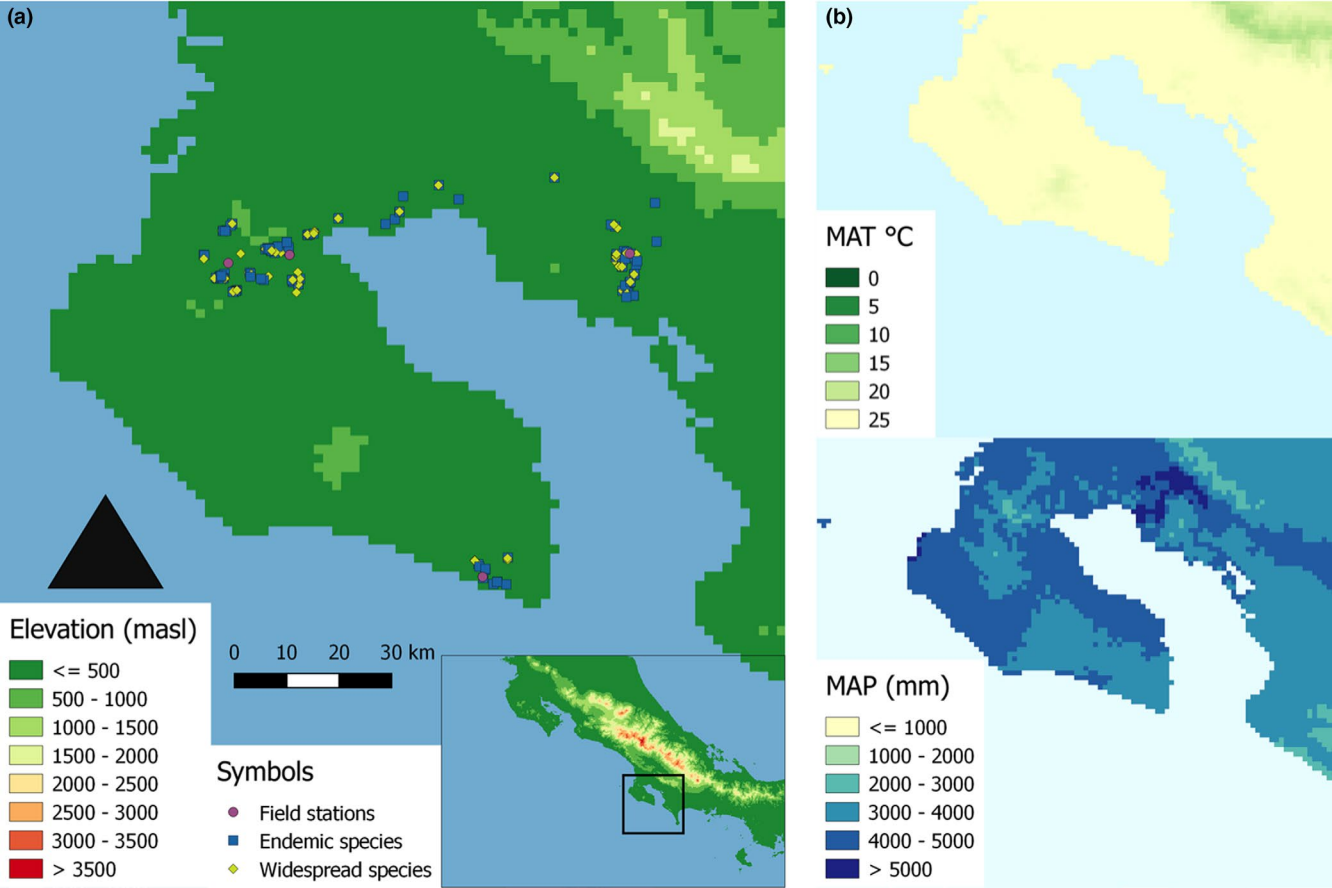
Study area and sampling sites of neotropical tree species in southwestern Costa Rica (Peninsula de Osa and Golfo Dulce). Colored points indicate locations of (1) field stations (purple), (2) endemic tropical tree species (blue), and (3) widespread congeners (yellow) surveyed for plant functional traits. Landscape heterogeneity in (a) topography, that is, elevation (in m a.s.l.) and (b) climate, that is, mean annual temperature (in °C) and mean annual precipitation (in mm) is displayed according to Hijmans et al. (2005). This figure was reproduced from (Chacón‐Madrigal et al., 2018a) according to Creative Commons Attribution 4.0 International Public License

**FIGURE 2:**
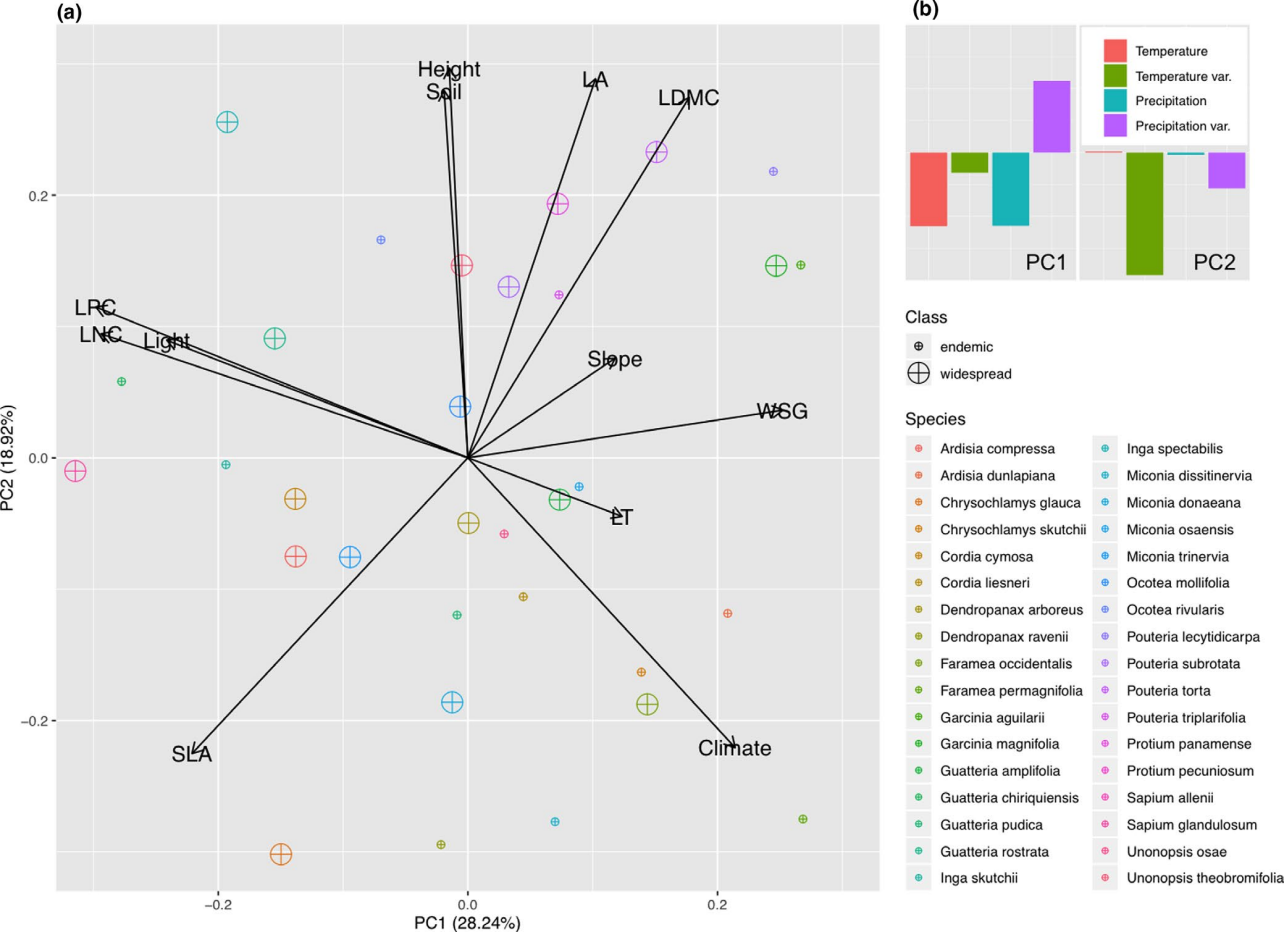
Principal component analysis (PCA) of eight plant functional traits—wood density (WSG), plant height (Height), specific leaf area (SLA), leaf area (LA), leaf thickness (LT), leaf dry‐matter content (LDMC), leaf nitrogen content (LNC), and leaf phosphorous content (LPC)—obtained from 335 tree individuals comprising 34 tree species (point color) classified into endemic and widespread species according to differences in range size (point size). Factor loadings reflect (a) in‐situ measurements, that is, microclimate (Climate), soil clay, sand, silt content (Soil), topography (Slope), and canopy‐light index (Light), as well as, (b) bioclimatic variables extracted from Worldclim, that is, temperature (red bar), temperature variation (green bar), precipitation (blue bar), and precipitation variation (purple bar)

We quantified relative amounts of variance observed within eight plant functional traits obtained from tropical tree individuals located in southwestern Costa Rica (Figure 1). Observed variation in plant functional traits ranged from 38.0 to 1645 cm^2^ for LA, from 0.16 to 0.61 mm for LT, from 66.4 to 236 g/cm^2^ for SLA, from 195 to 472 mg/g for LDMC, from 0.26 to 0.86 g/cm^3^ for WSG, from 1.17% to 3.07% for nitrogen content, and from 0.05 to 0.23 mg/g for phosphorus content (Table 1). A PCA investigating relationships between plant functional traits and environmental factors indicated that leaf traits varied in association with canopy light regime and soil nutrient content, whereas wood traits were related to topographic slope position and soil water content (Figure 2). Analyzing the underlying drivers of these relationships, we found that trait variation was relatively more strongly related to spatial distance, thus often masking trait variation in response to environmental factors due to autocorrelation of space and environment (Table 2).

**TABLE 2:** Results of multiple regression on distance matrices (MRM) showing significant relationships between distance matrices of the observed environmental factors (i.e., climate, soil, slope, light) and each of the plant functional traits

Component	Spatial variation	*p* value	Environmental variation	*p* value	Total variation	*p* value
	*R* ^2^		*R* ^2^		*R* ^2^	
Climate						
WSG	0.04	**0.02**	0.01	0.74	0.04	**0.04**
Height	0.01	0.54	0.00	0.77	0.01	0.60
SLA	0.01	0.12	0.00	0.13	0.02	0.23
LA	0.01	0.12	0.00	0.15	0.03	0.24
LT	0.01	0.24	0.00	0.65	0.01	0.54
LDMC	0.03	0.24	0.03	0.07	0.04	**0.03**
LNC	0.00	0.63	0.03	**0.05**	0.03	0.14
LPC	0.00	0.09	0.02	**0.03**	0.04	0.13
Soil						
WSG	0.03	**0.02**	0.00	0.32	0.04	0.06
Height	0.00	0.54	0.00	0.74	0.00	0.72
SLA	0.02	0.10	0.00	0.84	0.02	0.26
LA	0.03	0.09	0.01	0.41	0.03	0.18
LT	0.01	0.25	0.01	0.32	0.01	0.34
LDMC	0.01	0.23	0.00	0.74	0.01	0.42
LNC	0.00	0.62	0.00	0.68	0.00	0.81
LPC	0.02	0.11	0.00	0.57	0.02	0.22
Slope						
WSG	0.03	**0.03**	0.00	0.64	0.04	**0.04**
Height	0.00	0.53	0.01	0.29	0.01	0.52
SLA	0.02	0.11	0.00	0.73	0.02	0.22
LA	0.02	0.11	0.00	0.79	0.03	0.24
LT	0.01	0.24	0.00	0.52	0.01	0.41
LDMC	0.01	0.24	0.00	0.42	0.01	0.35
LNC	0.00	0.64	0.00	0.46	0.01	0.68
LPC	0.02	0.13	0.00	0.59	0.03	0.20
Light						
WSG	0.03	**0.02**	0.00	0.90	0.04	0.06
Height	0.01	0.54	0.01	0.29	0.01	0.38
SLA	0.02	0.12	0.00	0.80	0.02	0.22
LA	0.03	0.10	0.00	0.96	0.03	0.25
LT	0.01	0.25	0.01	0.25	0.02	0.30
LDMC	0.01	0.25	0.00	0.68	0.01	0.46
LNC	0.00	0.64	0.03	**0.03**	0.04	0.12
LPC	0.01	0.12	0.07	**0.01**	0.09	**0.04**

Note

Test statistics represent *R* ^2^ and *p* value (*p* < .05 highlighted in bold) showing significant relationships between environmental controlling factors and plant functional traits, while separating respective effects of nonplastic (correlation between trait distance matrix and spatial distance matrix while accounting for environmental variation), plastic (correlation between trait distance matrix and environmental distance matrix while accounting for spatial variation), and spatial components (correlation between geographic distance matrix and environmental distance matrix while correcting for trait variation).

Abbreviations: Ht, plant height; LA, leaf area; LMDC, leaf dry‐matter content; LNC, leaf nitrogen content; LPC, leaf phosphorous content; LT, leaf thickness; SLA, specific leaf area; WSG, wood density.

### 3.2 Trait variation due to spatial distance and environmental factors

**FIGURE 3:**
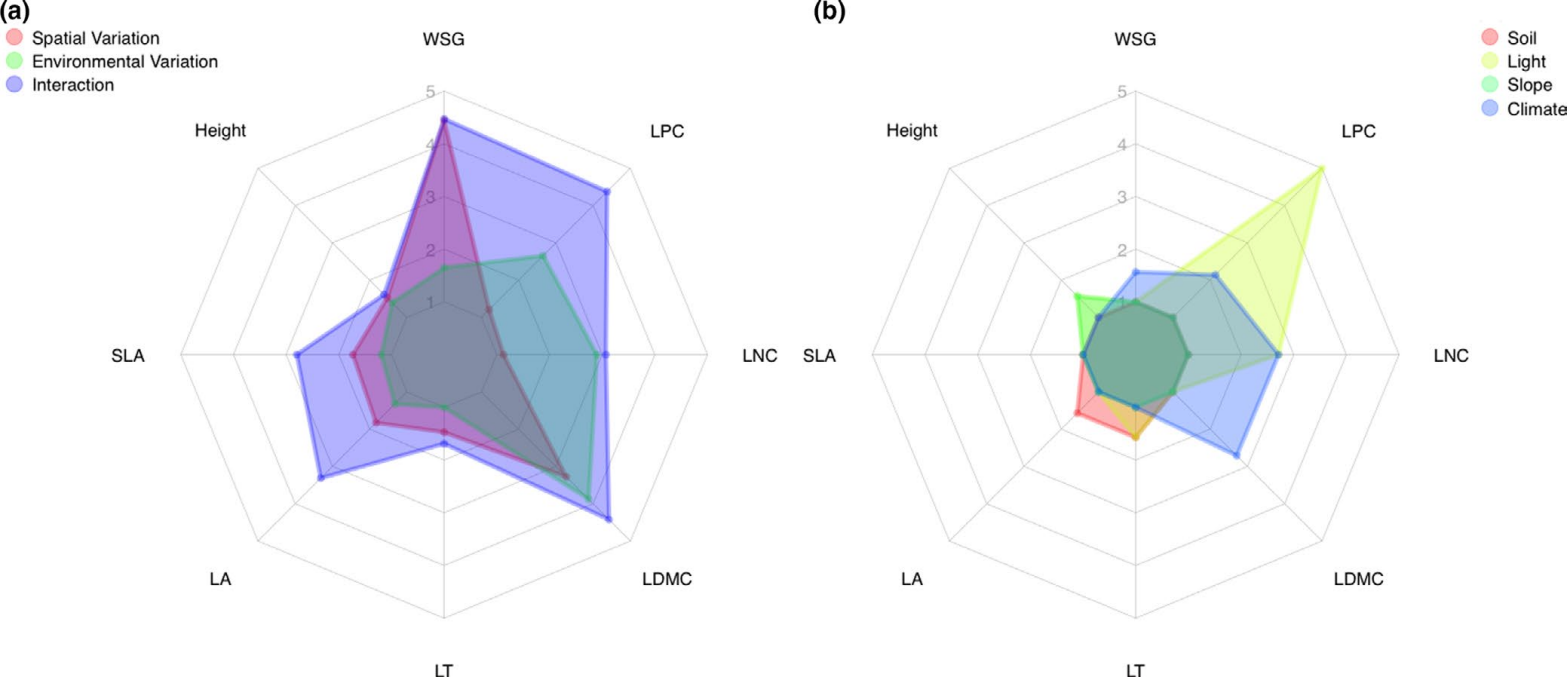
Radar plots displaying the relative amount of explained variance in multiple regression on distance matrices between respective components accounting for (a) spatial variation (red area), environmental variation (green area), and interaction between space and environment (blue area), as well as, for (b) environmental factors, such as soil texture “Soil” (red area), canopy‐light index “Light” (yellow area), slope position “Slope” (green area), and microclimate “Climate” (blue area), for each of the eight plant functional traits—wood specific gravity, i.e., wood density (WSG), plant height (Height), specific leaf area (SLA), leaf area (LA), leaf thickness (LT), leaf dry‐matter content (LMDC), leaf nitrogen content (N), and leaf phosphorous content (P) investigated in this study

We found that the relative amount of explained variation differed between the environmental and spatial components of trait variation identified in this study (Figure 3a). Our findings indicate that the relationship between wood density and spatial variation in soil texture (*p* = .02), slope inclination (*p* = .03), light availability (*p* = .02), and climatic drivers (*p* = .02) was primarily due to *spatial variation* in woody tissue between forest stands, whereas leaf tissue, as well as, leaf chemistry varied in response to *environmental factors*, such as light availability (*p* = .03 and *p* = .01, respectively) and microclimate (*p* = .03 and *p* = .01, respectively) (Table 2). Testing for the direct environmental drivers (Figure 3b) revealed that variation in wood density was mostly driven by precipitation (*p* = .01), temperature (*p* = .03), and light availability (*p* = .04), whereas leaf nitrogen content was mostly driven by precipitation (*p* = .04), and less so by soil nutrient availability (*p* = .05) and light availability (*p* = .07) (Table 3).

**TABLE 3:** Results of multiple linear regression showing the effects of environmental factors

Predictor	Intercept		Slope		Light		Soil		Temperature		Precipitation	
Response	*t‐*value	*p‐*value	*t‐*value	*p‐*value	*t‐*value	*p‐*value	*t‐*value	*p‐*value	*t‐*value	*p‐*value	*t‐*value	*p‐*value
WSG	−1.80	0.08	0.26	0.80	−2.15	**0.04**	−0.28	0.78	2.25	**0.03**	−2.84	**0.01**
Height	0.42	0.68	−0.17	0.87	1.86	*0.07*	1.18	0.25	−0.31	0.76	−0.02	0.99
SLA	0.94	0.35	0.13	0.90	0.69	0.50	−0.07	0.94	−0.84	0.41	0.73	0.47
LA	0.07	0.94	0.73	0.47	−1.04	0.31	0.92	0.36	−0.08	0.94	0.37	0.71
LT	1.17	0.25	−0.01	1.00	0.37	0.71	−0.91	0.37	−1.03	0.31	0.84	0.41
LDMC	−2.26	**0.03**	0.07	0.94	−1.14	0.26	1.26	0.22	2.68	**0.01**	−3.06	**0.00**
LNC	0.94	0.35	−0.64	0.53	1.86	*0.07*	2.02	*0.05*	−1.08	0.29	2.18	**0.04**
LPC	0.57	0.58	−0.93	0.36	1.99	*0.06*	0.73	0.47	−0.81	0.43	1.96	*0.06*

Note

Slope, slope position (Slope), canopy‐light index (Light), soil texture (Soil), temperature (Temperature), and rainfall (Precipitation)—on the variation in eight plant functional traits. Italic entries represent test statistics, such as *t*‐value (coefficients divided by standard errors) and *p*‐value (indicating significant relationships *p* < .05 in bold). Test statistics represent *t‐*value (coefficients divided by standard errors) and *p‐*value, showing significant relationships (*p* < .05 highlighted in bold) between plant functional traits and each of the environmental controlling factors.

Abbreviations: Height, plant height; LA, leaf area; LMDC, leaf dry‐matter content; LT, leaf thickness; LNC, leaf nitrogen content; LPC, leaf phosphorus content; SLA, specific leaf area; WSG, wood specific gravity, i.e., wood density.

### 3.3 Trait variation due to plant life‐history strategy and taxonomic species diversity

**FIGURE 4:**
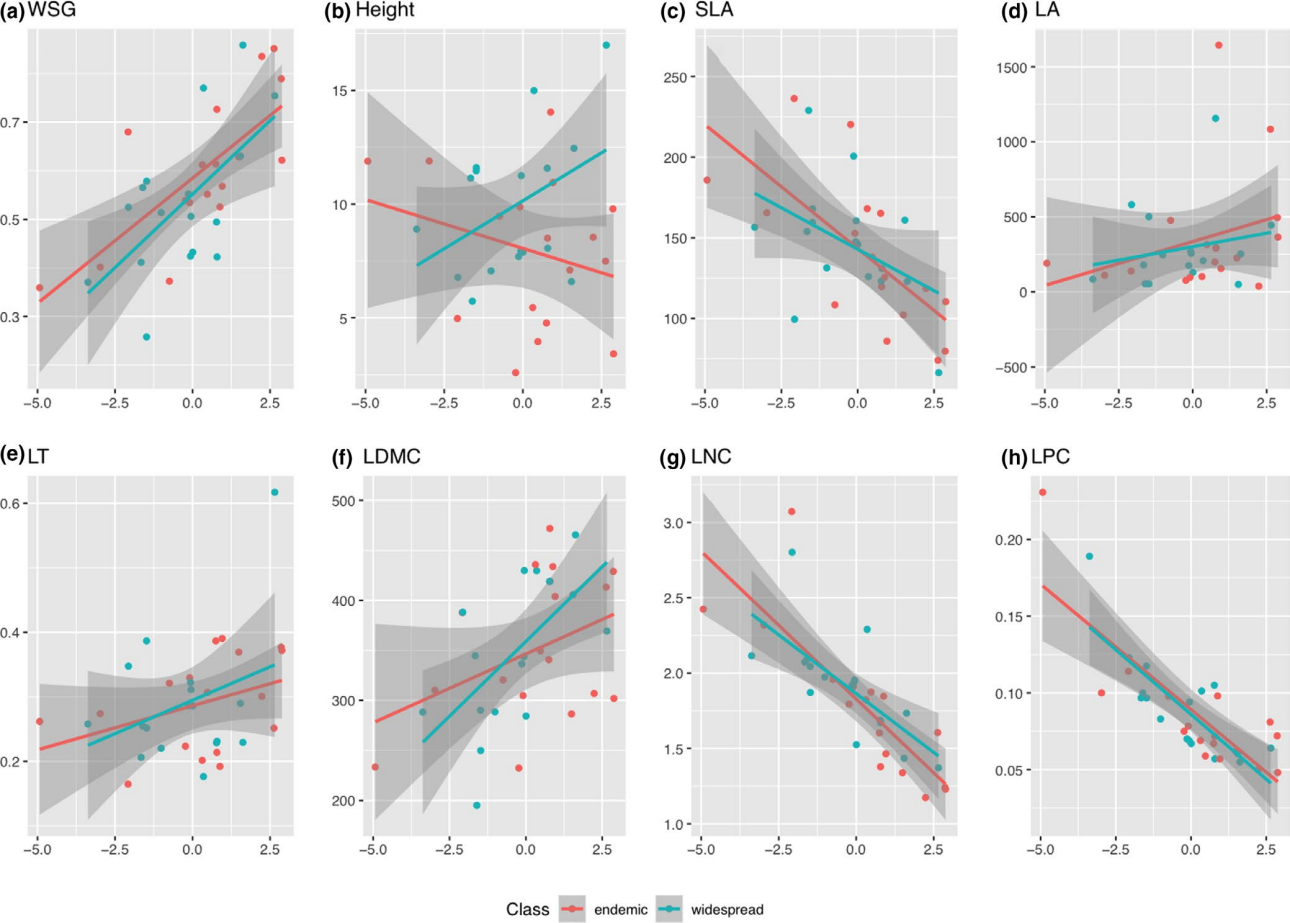
Scatterplots depicting the functional response of endemic (red points and regression line) and widespread (green points and regression line) tropical tree species to factors loadings of the first principal component of environmental factors (i.e., increasing temperature and precipitation variation as presented in Figure 2b), for each of the eight plant functional traits—(a) wood sepcific gravity, i.e., wood density (WSG), (b) plant height (Height), (c) specific leaf area (SLA), (d) leaf area (LA), (e) leaf thickness (LT), (f) leaf dry‐matter content (LMDC), (g) leaf nitrogen content (LNC), and (h) leaf phosphorous content (LPC) investigated in this study

**FIGURE 5:**
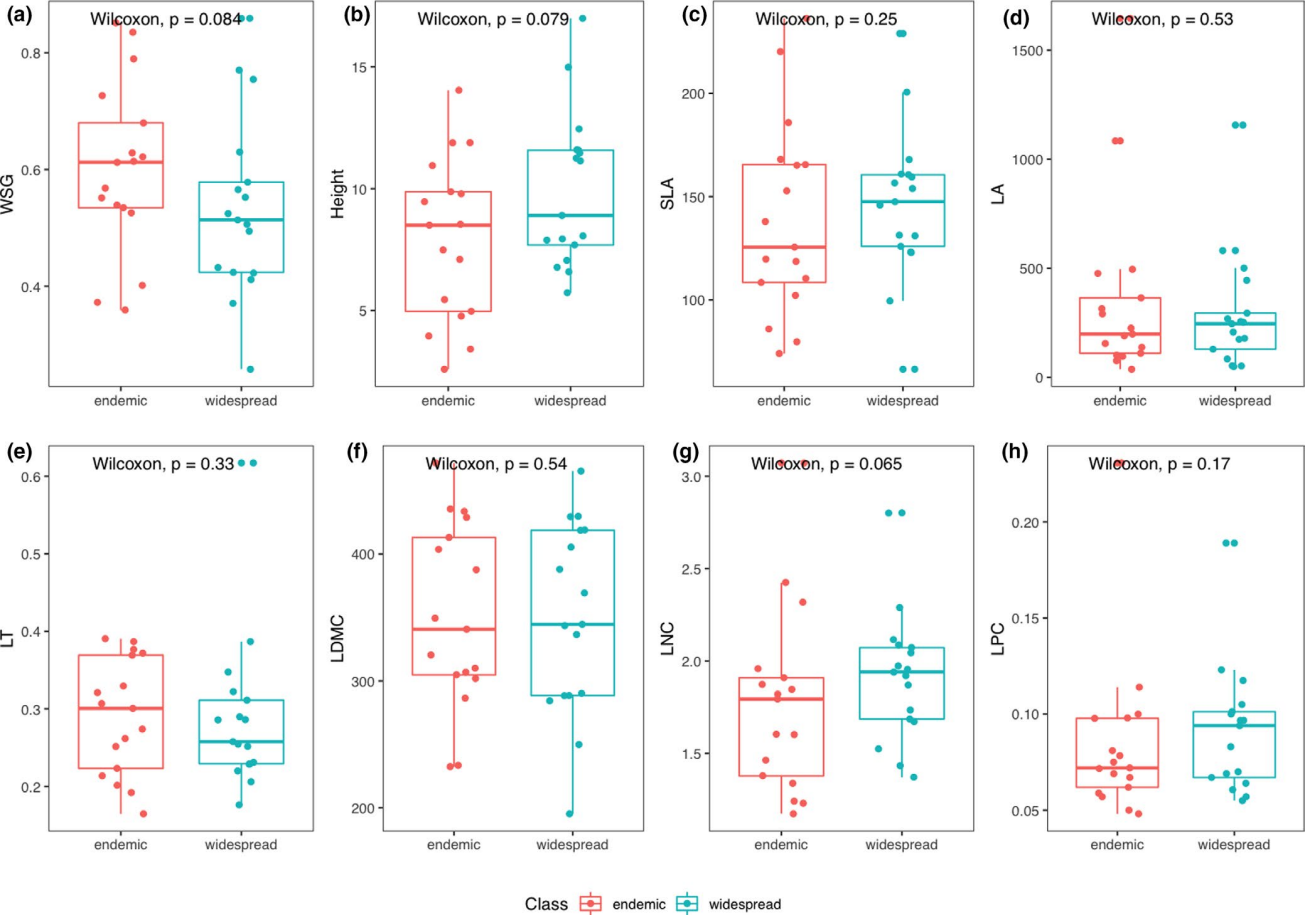
Boxplots indicating differences between endemic (red dots and boxes) and widespread (green dots and boxes) tropical tree species for each of the eight plant functional traits—(a) wood specific gracity, i.e., wood density (WSG), (b) plant height (Height), (c) specific leaf area (SLA), (d) leaf area (LA), (e) leaf thickness (LT), (f) leaf dry‐matter content (LMDC), (g) leaf nitrogen content (LNC), and (h) leaf phosphorous content (LPC) investigated in this study. Test statistics indicate significant differences between endemic and widespread species, based on Wilcoxon rank‐sum test and *p* values

**FIGURE 6:**
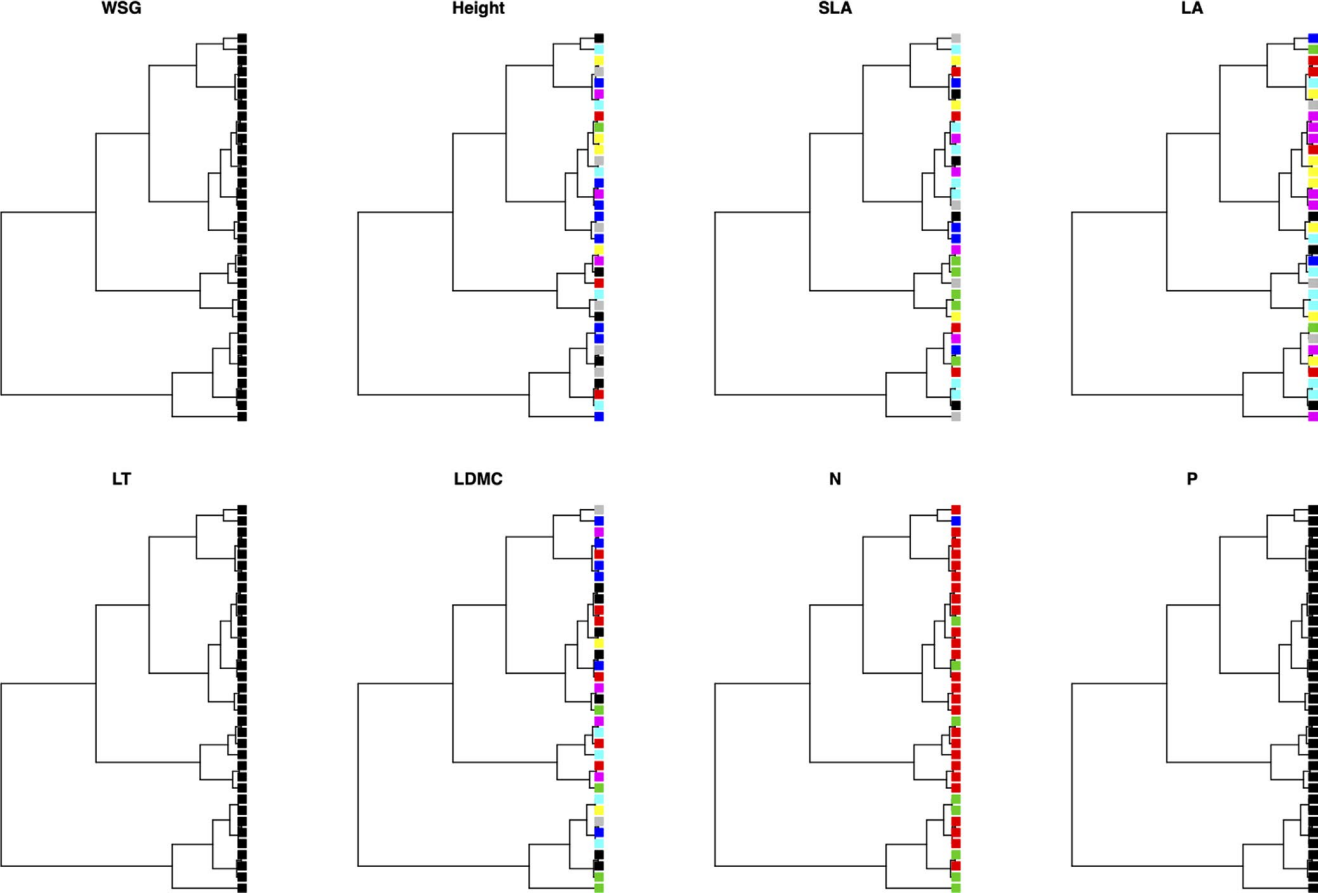
Taxonomic dendrogram depicting phylogenetic constraints on trait variance for each of the eight plant functional traits, that is, wood specific gravity, i.e., wood density (WSG), plant height (Height), specific leaf area (SLA), leaf area (LA), leaf thickness (LT), leaf dry‐matter content (LMDC), leaf nitrogen content (LNC), leaf phosphorous content (LPC) investigated in this study. Branch node color indicates a phylogenetically conserved signal among the nodes for 34 tropical tree species). For information about the tree species, please see Table 1

We further found differences in plant functional reaction norms to bioclimatic controlling factors (i.e., slopes of trait response versus. environmental variation) between endemic and widespread tropical tree species, when plotting each plant functional trait against the principal component of the extracted bioclimatic variables (Figure 4). Although we did not find strict significant differences (*p* < .05) in trait variation between endemic and widespread tropical tree species, we found that endemic species tended to exhibit higher wood density (*p* = .08), smaller tree size (*p* = .08), and higher leaf nitrogen content (*p* = .07) compared to widespread tropical tree species (Figure 5), which might reflect differences in plant life‐history strategy between endemic and widespread tropical tree species. Eventually, we found a significant relationship between phylogenetic distance and functional trait variance due to taxonomic relatedness of the sampled tree individuals (belonging to congeneric pairs of widespread and endemic tree species), such that a clear phylogenetic pattern was found for tree height, SLA, LA, LDMC, and LNC, whereas such pattern was missing for WSG and LPC (Figure 6).

## 4 DISCUSSION

We applied a statistical technique accounting for multiple and interrelated components of plant functional trait variation by partitioning total observed variation into components uniquely and jointly explained by environmental heterogeneity, and spatial distance between sampling sites. We found (i) significant interactions between spatial distance and environmental controlling factors, (ii) different environmental controls across plant tissues and associated plant functional traits, and (iii) nonuniform functional responses among coexisting tropical tree species. We conclude that our current understanding of tropical ecosystem functioning in response to projected climate change would benefit from accounting for the underlying mechanisms driving plant functional trait variation in tropical forests.

### 4.1 Controls over plant functional trait variation in tropical forests

We found that plant functional trait variation is the product of multiple mechanisms and different drivers, including climate but also topoedaphic factors and biotic interactions. In line with our findings, it has been reported that trade‐offs at the species level were only weakly associated with climate and soil conditions when analyzing global trait‐environment relationships at the global scale (Bruelheide et al., 2018), because trait combinations were predominantly filtered by local‐scale factors such as disturbance, fine‐scale soil conditions, niche partitioning, and biotic interactions (Grime, 2006). However, because both biotic and abiotic factors do not mutually exclusively affect trait variation, and usually shift in their relative dominance over trait expression across spatial gradients in response to multiple environmental factors, ideally all of these factors should be accounted for when analyzing plant functional trait variation. Here, we found that all of the plant functional traits investigated in this study varied with both spatial distance and environmental factors and therefore applied a statistical method to decompose respective components driving trait variation in response to multiple environmental factors, that is, soil texture, canopy‐light index, slope position, temperature, and rainfall (Figure 3).

### 4.2 Plant functional trait variation in response to environmental factors and spatial distance

Despite a relatively large amount of unexplained variation due to factors not accounted for in the analysis (see *R^2^* values in Table 2), we were able to identify plant functional trait variation in response to environmental heterogeneity among, and spatial distance between sampling sites. Recalling our assumption about respective components of trait variation, the intra‐specific component due to phenotypic plasticity between individuals of one species would be driven by the heterogeneity of the local environment, independent from spatial factors, whereas the inter‐specific component due to genetic adaptation and species turnover would be expected to increase with geographic distance between forest stands. Most strikingly, we found this pattern reflected among different plant tissues, such that wood traits varied in response to the spatial component and thus appear less plastic, while leaf traits were more related to the environmental component and thus appear more plastic (Figure 3), both of which would be in line with the proposed trade‐offs along the plant‐economics spectrum (Reich, 2014).

### 4.3 Plant functional trait variation and the plant‐economics spectrum

Our results, highlighting differences in the strength of relationships between respective components and plant tissues, mirror the underlying mechanisms driving trade‐offs in relative investment between canopy and woody tissues in response to multiple limiting factors (Townsend et al., 2008). We found that leaf nitrogen content and leaf phosphorous content was related to canopy‐light regime, while wood density and plant height was associated with slope position and soil texture (Figure 2). Our results indicate that short‐term eco‐physiological responses at the canopy‐level or leaf‐level are associated with canopy‐light regime, whereas rather longer‐term investments into woody tissue are related to topoedaphic and climatic factors (Figure 3). Overall, this confirms our assumption that plant functional trait variation is controlled by multiple mechanisms and interrelated driving factors, and our findings of trade‐offs in relative investment between canopy and woody tissues furthermore indicate that along environmental gradients of resource availability species should be filtered according to differences in their life‐history strategy.

### 4.4 Plant functional traits and species composition across environmental gradients

Our analysis revealed differences in the functional response among coexisting neotropical tree species, which suggests that under projected climate change range‐restricted endemic species might be more susceptible to competitive exclusion than more widespread congeners (Figure 4). Such a differential response of neotropical tree species to climate change has been reported in a study indicating a shift to more dry‐affiliated taxa across Amazonia, where tree communities have become increasingly dominated by large‐statured pioneers, while short‐statured taxa decreased over the observation period (Esquivel‐Muelbert et al., 2019). Indeed, we here found that endemic species were on average characterized by higher wood density and lower leaf nitrogen content compared to their widespread congeners (Figure 5). Our findings are in line with a foregoing analysis conducted in the same study region, which found that range‐restricted species with conservative ecological strategies were characterized by high wood density and low leaf nitrogen content, in comparison to coexisting but more widespread species (Chacón‐Madrigal et al., 2018b). Hence, the observed differences in plant functional traits between coexisting widespread and congeneric endemic tree species might trigger differences in the functional response of tropical plant communities due to differences in their eco‐evolutionary trajectory and associated ecological life‐history strategy.

According to life‐history theory, the physical and chemical properties of forest soils determining forest structure and dynamics across the Amazon Basin (Quesada et al., 2012) shape plant‐community composition by differentially favoring species depending on their life‐history strategy (Oliveira et al., 2018). In particular, while relatively stable environments on flat terrain with high clay content and low nutrient availability favor slow‐growing tree species, more frequently disturbed environments on steep terrain with low clay content and high nutrient availability favor fast‐growing tree species competing for limiting resources (Werner & Homeier, 2015). Accordingly, it has been found that tropical plant species composition was strongly related to local topoedaphic factors affecting resource availability (Hofhansl et al., 2020), which furthermore determined the climate sensitivity of neotropical tree species (Hofhansl et al., 2014). Hence, the opposed functional responses between coexisting neotropical tree species found in this study might reflect differences in their ability to compete for limiting resources, thus suggesting that endemic species might be prone to competitive exclusion under projected climate change.

### 4.5 Implications for trait‐based vegetation models

So far, it has remained elusive to what extent the available information on trait variance and trade‐offs in life‐history strategy among coexisting species can be used to derive mathematical models capable of reliably predicting future ecosystem functioning. On the one hand, studies exploring plant functional traits have suggested that a classification based on trait co‐variations should be a powerful candidate for building a new generation of vegetation models capable of predicting the response of vegetation to future climate changes (Zhao, 2019). On the other hand, studies have found that trait variation was not predictable because factors other than climate, such as site conditions, growth form, and phylogeny were important determinants of the observed trait variation (Yang et al., 2018). Accordingly, a trait‐based forest model exploring the relative roles of climate and plant traits in controlling forest productivity and structure found that, while differences in productivity were driven by climate, demographic rates, such as mortality and recruitment, were linked to plant traits (Fauset et al., 2019). These findings are in line with our observation that multiple and interrelated factors determined plant functional trait variation in tropical forests; however, our results also indicated that most of the variation in plant functional traits could not be explained by the comprehensive set of environmental factors analyzed in this study. Potentially, some of this variation could be accounted for by other quantifiable, deterministic factors; but our findings (of relatively large amounts of unexplained trait variation) suggest that interactive effects and nondeterministic factors are of similar importance, which would imply that spatial autocorrelation and stochasticity should be accounted for in next‐generation approaches. Recently, some studies have proposed novel concepts based on multi‐dimensional hypervolume (Blonder et al., 2014), trait probability density (Carmona et al., 2016), and the biochemical niche (Peñuelas et al., 2019), thus allowing to more realistically assess plant functional responses of hyper‐diverse ecosystems to climate change (Bartlett et al., 2018). Implementation of the findings presented in this study allows to account for different components of trait variation, which should improve predictions of plant functional response spectra to environmental variation and therefore result in more reliably projections of ecosystem functioning under future scenarios (Franklin et al., 2020).

## CONFLICT OF INTEREST

The authors have no conflicts of interest to declare.

## AUTHOR CONTRIBUTION

**Florian Hofhansl:** Conceptualization (lead); Writing‐original draft (lead); Writing‐review & editing (lead). **Eduardo Chacon:** Conceptualization (supporting); Data curation (lead); Investigation (lead); Resources (lead); Validation (lead); Writing‐original draft (supporting); Writing‐review & editing (supporting). **Ake Brännström:** Conceptualization (supporting); Validation (supporting); Writing‐review & editing (supporting). **Ulf Dieckmann:** Methodology (supporting); Supervision (supporting); Writing‐review & editing (supporting). **Oskar Franklin:** Conceptualization (supporting); Supervision (lead); Writing‐original draft (supporting); Writing‐review & editing (supporting).

## Data Availability

Data used in this analysis have been deposited in the Plant Trait Database (https://www.try‐db.org/TryWeb/Home.php) available under the following link: https://doi.org/10.17871/TRY.12 (https://www.try‐db.org/TryWeb/Data.php#12).
